# A review of research progress of mirror therapy in peripheral nerve injury

**DOI:** 10.3389/fneur.2026.1740020

**Published:** 2026-03-09

**Authors:** Chengqi Duan, Hongxia Gan, Xiujian Zhuo, Yanping Xiao, Xiaobo Chen

**Affiliations:** 1 School of Rehabilitation, Gannan Medical University, Ganzhou, China; 2 Department of Rehabilitation Medicine, The Second Affiliated Hospital of Gannan Medical University, Ganzhou, China; 3 Department of Rehabilitation Medicine, The First Affiliated Hospital of Gannan Medical University, Ganzhou, China

**Keywords:** mirror neuron system, mirror therapy, nerve regeneration, neuroplasticity, peripheral nerve injury

## Abstract

**Background:**

Mirror therapy, as a novel rehabilitation approach, has been widely applied in the treatment of various diseases, including post-stroke limb dysfunction, unilateral neglect, peripheral facial paralysis, complex regional pain syndrome, and childhood cerebral palsy.

**Purpose:**

This study aims to investigate the effectiveness of mirror therapy in treating peripheral nerve injuries, expand the scope of diseases to which mirror therapy can be applied, and provide a novel treatment strategy for peripheral nerve injuries.

**Methods:**

This study conducted a comprehensive search and analysis of relevant clinical research on mirror therapy for peripheral nerve injuries, providing theoretical and clinical basis for the application of mirror therapy in the treatment of peripheral nerve injuries.

**Results:**

Based on a comprehensive analysis of seven clinical studies, it can be concluded that mirror therapy (MT) has a positive effect on improving motor function.

**Conclusion:**

Mirror therapy is characterized by its simplicity, economy and non-invasiveness. It is an effective rehabilitation technique for treating peripheral nerve injuries and is suitable for low-cost, long-term home-based rehabilitation. It can be combined with conventional therapies to jointly promote the recovery of motor functions and accelerate the plastic regeneration of nerves.

## Introduction

Peripheral nerve injury (PNI) is usually caused by damage to the patient’s peripheral nerve plexus, nerve trunk or its branches due to external forces, such as traction, cutting, compression, firearm injury, etc. A survey of Canadian researchers showed that the annual incidence of neurological damage in trauma populations ranged from 0.8 to 3.6% and was more common in young men (1). The mean incidence of PNI in the United Kingdom was 11.2 (95% CI 10.9, 11.6) events per 100,000 persons per year (2). In the United States, 2.6% of post-traumatic patients were diagnosed with upper extremity nerve injuries, while 1.2% were diagnosed with lower extremity nerve injuries (3). Among these cases, a majority (90.8%) presented with a single nerve injury, whereas 8.2% had two distinct nerves affected and only 1% exhibited damage to more than two nerves. In China, there are approximately 600,000 to 900,000 patients with peripheral nerve injuries each year (4). An epidemiological study on upper extremity peripheral nerve injuries in South Korea from 2008 to 2018 indicated that the most common peripheral nerve injuries in the limbs were the ulnar nerve, median nerve, radial nerve, sciatic nerve, and common peroneal nerve. Upper extremity nerve injuries accounted for 60–70% of all cases. Among peripheral nerve injuries (PNI), digital nerve injuries were the most frequent, followed by radial nerve, ulnar nerve, and median nerve injuries (5).

The symptoms of patients with peripheral nerve injury include motor dysfunction, sensory dysfunction, neuropathic pain, abnormal sweating and so on. Due to the slow rate of nerve regeneration and the loss of neurotrophic effects, patients with peripheral nerve injuries often experience muscle atrophy, joint stiffness, and are likely to develop long-term complications. These issues severely impact patients’ physical activity and quality of life. Analysis of the National (nationwide) Inpatient Sample (NIS) by Karsy et al. (6) revealed distinct epidemiological and economic burdens of PNI. The annual incidence was estimated at 43.8 per million for upper extremity PNI and 13.3 per million for lower extremity PNI. The associated median hospitalization costs ranged from $47,000 to $64,000 with a compound annual growth rate (CAGR) exceeding 8% (7). These costs are primarily driven by surgical interventions, inpatient rehabilitation, and complication management. In addition to direct medical expenditures, the disease imposes considerable indirect economic burdens. It is estimated that the lifetime indirect cost per patient with traumatic brachial plexus injury amounts to $868,000 exerting profound economic impacts on both individuals and society at large (8).

## Mirror therapy

### Overview of mirror therapy

Mirror therapy, also referred to as mirror visual feedback therapy, is a rehabilitation technique based on the principle of mirror reflection. A mirror is placed between the bilateral limbs, allowing the patient to move the unaffected limb while observing its reflected image. This approach acts by modulating central brain perception through integrated visual feedback, motor imagery, and visual illusion. The induced illusion of normal limb movement modulates sensorimotor function, resulting in enhanced motor performance and facilitating functional reorganization of the brain.

In 1995, Ramachandran et al. (9) first proposed mirror therapy (MT) to treat phantom limb pain in amputees, achieved remarkable efficacy. In 1999, Altschuler et al. (10) applied MT to address upper limb motor dysfunction post-stroke. In 2007, Sütbeyaz et al. (11) extended its use to treat lower limb motor dysfunction after stroke. Subsequently, an increasing number of researchers have conducted clinical studies in this field, expanding its application to conditions such as unilateral neglect, peripheral facial palsy, complex regional pain syndrome (CRPS), and pediatric cerebral palsy (12–16). Rehabilitation techniques derived from mirror therapy, such as action observation therapy and motor imagery therapy, have been widely adopted in clinical practice (17–19).

While traditional mirror therapy, which involves placing a mirror between a patient’s limbs to observe reflected movements, is a recognized technique, its effectiveness is heavily dependent on both equipment quality and patient initiative. With technological advances, modernized mirror therapy utilizes imaging technology to project movements onto computer screens. Through image reversal, patients gain intuitively visual feedback of their “affected limb” in motion. This enhances training motivation and participation, thereby improving therapeutic outcomes. Furthermore, technological advances in virtual reality (VR) and brain-computer interfaces (BCI) have spurred new developments in mirror therapy (18).

### The mechanism and application of mirror therapy

The predominant hypothesis regarding the mechanism of mirror therapy posits that it activates mirror neurons, promotes functional reorganization in the brain, and facilitates the recovery of limb motor function. In 1996, Rizzolatti et al. (20) observed neuronal firing in the F5 region of the premotor cortex during movement observation experiments with macaque monkeys. They identified these neurons as “mirror neurons” (MNS), which are now known to be closely associated with action observation-execution matching mechanisms.

As research progressed, subsequent studies confirmed the existence of MNS in the human brain (21–23). The human MNS comprises a complex neuronal network distributed across multiple cortical regions. Contemporary research divides this system into two components: (1) The parieto-frontal mirror system, comprising the ventral premotor cortex, inferior precentral gyrus, rostral inferior parietal lobule, posterior inferior frontal gyrus, and middle temporal gyrus; (2) The limbic mirror system, involving the anterior cingulate cortex, amygdala, insula, and prefrontal cortex, among others (24).

MNS not only matches observed actions with executed actions but is also associated with diverse human functions. MNS in different brain regions serve distinct roles, playing crucial parts in action observation-execution matching, action imitation, motor learning, motor understanding, and emotion recognition (25).

Therefore, due to the extensive influence of MNS activation on cortical brain function, the application fields of MT are also highly diverse. Clinical randomized controlled trial (RCT) results from Mao et al. (26) demonstrate that training based on the MNS combined with conventional training can improve upper limb motor function and cognitive function in stroke patients. Functional magnetic resonance imaging (fMRI) revealed significantly heightened activation of the MNS in children with unilateral cerebral palsy compared to typically developing controls during observation of grasping actions. This finding suggests that action observation therapy based on the mirror neuron system may facilitate functional improvements in this patient population (27). One study utilized hand-action observation training based on mirror neuron theory to treat aphasia patients, analyzing changes in brain activation during action observation (28). The results demonstrated that compared to observing dynamic objects, hand-action observation appears to activate the mirror neuron system more robustly. Combining hand-action observation with repetitive practice may better improve language function in aphasia patients. Wang et al. (29) proposed that inducing mirror neuron activation in stroke patients can reactivate the top-down swallowing neural network, thereby improving dysphagia and enhancing the quality of life for stroke survivors. For Parkinson’s patients with gait disorders, a systematic review found that visuomotor training—action observation and motor imagery—improves walking, yielding benefits in overall disease severity, balance, and quality of life (30).

## Mirror therapy for peripheral nerve injury

### Strategy of literature search

*Deduplication*: Duplicate records were removed using reference management software (EndNote 20) and manual verification.

*Initial screening*: Based on titles and abstracts, the following inclusion criteria were applied:

1) The study topic involved mirror therapy and peripheral nerve injury.
2) The study design was a randomized controlled trial or a clinical controlled trial.
3) Mirror therapy served as the primary intervention or a significant component of a combined treatment regimen.

*Full-text screening*: Full texts of studies passing the initial screen were retrieved and reviewed against the same inclusion criteria. The following exclusion criteria were simultaneously applied:

1) Unable to access the full text or data is missing.
2) Publication types such as reviews, meta-analyses, commentaries, conference abstracts, study protocols, or other non-original clinical research.
3) Study populations with non-peripheral nerve injuries.

**Figure 1:**
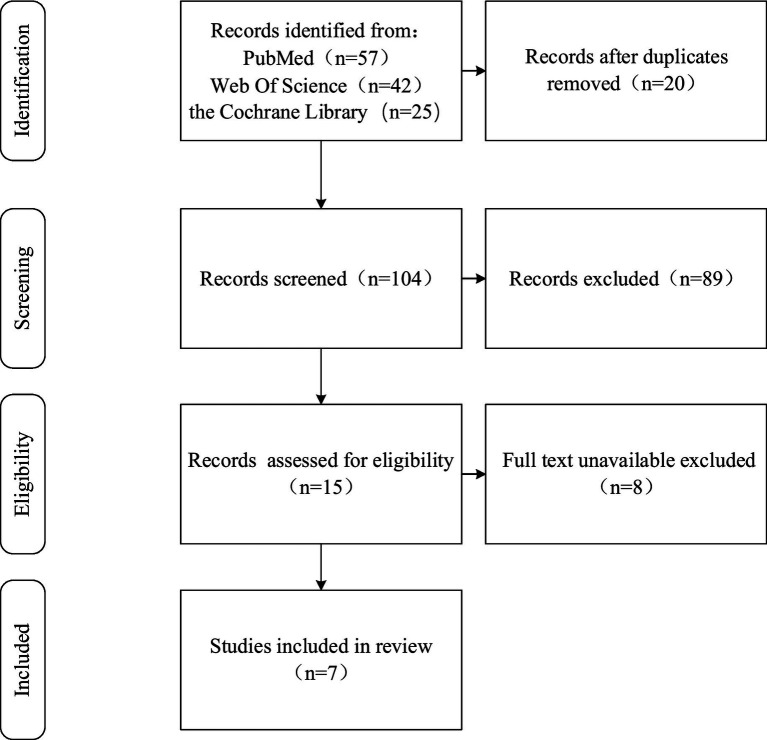
Flowchart of literature screening.

The literature obtained after screening was classified and organized through full-text reading to derive the following Figure 1 and Table 1.

**Table 1:** Characteristics of the study.

Author	Population	Intervention duration	Intervention	Assessment schedule and outcome measures	Conclusions
Paula et al. (31)	Patients following surgical repair of hand nerve injuries (*n* = 20)	6 weeks, 3 times per week	Research group: 30 min early sensory re-education and 30 min home mirror therapy (started 1 week after surgery) Control group: 30 min late-stage sensory re-education (started 3–5 months after surgery)	Time: Before intervention, 3 months after intervention, 6 months after intervention Assessment tools: Rosen score, Semmes–Weinstein test (SWM), Disabilities of the Arm, Shoulder and Hand (DASH) questionnaire	MT provided no benefit over traditional re-education
Hsu et al. (34)	Patients following surgical repair of hand nerve injuries (*n* = 12)	12 weeks, 3 times per week	Research group: 15 min mirror therapy, 20 min regular hand therapy and 20 min physical therapy Control group: 15 min protective/discriminative sensory re-education, 20 min conventional hand therapy and 20 min physical therapy	Time: Before intervention, 12 months after intervention, follow-up after 12 weeks Assessment tools: SWM test, two-point discrimination test (2-PD), Purdue pegboard test (PPT), Minnesota Manual Dexterity Test (MMDT), and pinch activity test	MT was more significant improvement in fine hand function, but no significant difference from control in monofilament test
Chen et al. (33)	Patients following surgical repair of hand nerve injuries (*n* = 6)	12 weeks, 2 times per week	Research group: 60 min conventional PT: edema control, scar massage, electrical stimulation for denervated muscle, ROM, and strength training; +30 min MT Control group: 60 min conventional PT + 30 min sensory re-education	Time: Before intervention, post-intervention (immediate), 12 weeks after intervention Assessment tools: SWM test, static two-point discrimination test (S-2PD), grip strength, DASH questionnaire	The MT group showed better outcomes in finger dexterity
Kablanoğlu et al. (32)	Patients following surgical repair of hand nerve injuries (*n* = 20)	6 weeks, 5 times per week	Research group: 45 min conventional PT + 15 min MT Control group: 45 min conventional PT	Time: Before intervention, 6 weeks after intervention Assessment tools: Visual Analog Scale (VAS), Duruöz Hand Index (DHI), DASH questionnaire, Jebsen Hand Function Test (JHFT), SWM test	Mirror therapy significantly improves fine hand functions
Chu et al. (35)	Patients with common peroneal nerve injury (*n* = 30)	4 weeks, 4 times per week	Research group: 30 min neuromuscular electrical stimulation (2 Hz, square wave, 30–40 mA, at maximum patient tolerated intensity) + 30 min MT Control group: 30 min neuromuscular electrical stimulation	Time: Before intervention, 4 weeks after intervention Assessment tools: Surface electromyography testing, range of motion, monofilament testing, vibration sense testing, humidity	Mirror therapy combined with neuromuscular electrical stimulation demonstrated significant therapeutic effects on superficial sensation, nerve conduction velocity, and range of motion in patients with peroneal nerve injury
Paolucci et al. (36)	Patients with peripheral facial palsy	12 weeks, 2 times per week	Research group: 30 min conventional rehab (facial expression therapy & myofascial techniques) + 30 min MT & motor imagery (MI) Control group: 30 min conventional rehab	Time: Before intervention, 1 month after intervention, 2 months after intervention, 3 months after intervention, and follow-up after 2 months Assessment tools: House–Brackmann Scale, Facial Disability Index (FaCE) Scale, Emotional and Depressive Status	MT&MI group showed significant improvements in facial symmetry and motor function, quality of life, and depressive symptoms
Martineau et al. (37)	Patients with acute Bell’s palsy	12 weeks, four clinical sessions in the first two weeks, followed by two daily sessions at home	Research group: MT + MI + manipulations Control group: consultation and guidance	Time: 10 to 14 days after the onset, and 1, 2, 3, 4, 5, 6, and 12 months after the onset Assessment tools: Facial symmetry (House–Brackmann 2.0 grading, House–Brackmann 2.0 score and Sunnybrook score), coordinated movement, quality of life, perceived speech intelligibility	MT&MI group showed significant improvements in facial symmetry, coordinated movement, quality of life

### Peripheral nerve injury of the limbs

A randomized controlled trial investigating early sensory re-education of the hand through mirror therapy after peripheral nerve repair surgery included 20 patients with repaired hand nerves. Participants received either early postoperative mirror therapy or conventional late-stage sensory re-education training. The study concluded that mirror therapy provided no benefit over traditional re-education (31). This result challenges the widely accepted rehabilitation principle that interventions should be implemented promptly following nerve repair surgery. Another randomized controlled trial involving patients with peripheral nerve injuries in the hand randomly assigned 26 participants to either a mirror therapy group or a control group (32). Post-treatment assessments of pain, hand function, and sensation were conducted using a dynamometer, the Visual Analog Scale (VAS), the Duruöz Hand Index, the Quick Disabilities of the Arm, Shoulder, and Hand (Quick DASH) questionnaire, the Jebsen Hand Function Test, and Semmes-Weinstein monofilament testing. The results indicated that the mirror therapy group demonstrated greater improvement in hand skill function compared to the control group. The researchers concluded that the combination of mirror therapy with conventional rehabilitation may offer additional benefits for the recovery of hand function in patients with peripheral nerve injuries. Chen et al. (33) administered mirror therapy immediately following peripheral nerve repair in the forearm. They found that the mirror therapy group achieved superior outcomes in both finger dexterity and manual dexterity compared to the sensorimotor training group. Furthermore, fMRI revealed increased activation levels in corresponding brain regions during motor tasks. In a study conducted by Hsu et al. (34), tactile observation combined with task-based mirror therapy was applied to patients following hand nerve surgery. Their results indicated greater improvements in hand motor control assessments within the mirror therapy group.

In contrast to its application for peripheral nerve injuries in upper limbs, research on mirror therapy for lower limb peripheral nerve injuries is considerably limited. A recent study by Chu et al. (35) integrated neuromuscular electrical stimulation with mirror therapy to treat patients with common peroneal nerve injury. The findings demonstrated that after 4 weeks of neuromuscular electrical stimulation combined with mirror therapy, significantly improved outcomes were observed compared to simple electrical stimulation therapy alone, particularly in superficial sensation, nerve conduction velocity, and range of motion (ROM).

### Peripheral facial palsy

A double-blind randomized controlled trial conducted by Paolucci et al. (36) integrated MT and motor imagery (MI) with conventional rehabilitation to assess their therapeutic efficacy in patients with peripheral facial palsy. The results indicated superior outcomes in the experimental group, particularly in House–Brackmann scale scores, quality of life, and depressive symptoms. In a randomized controlled trial on the “Mirror Effect Plus Protocol” for treating acute Bell’s palsy, the researchers conducted a 1-year follow-up. The results showed that MEPP significantly improved facial symmetry, synkinesis, and quality of life in patients with acute Bell’s palsy (37).

### Comprehensive analysis

Based on a comprehensive analysis of seven clinical trials, most studies have reported that MT has a positive effect in improving motor function and has positive value in promoting neural plasticity. The application characteristics of mirror therapy are diverse, with intervention timing ranging from early postoperative period to the middle stage, and intervention duration ranging from 4 weeks to 12 months. The treatment frequency is most commonly 2–3 times per week, and the duration of each treatment is usually 15–30 min. Mirror therapy is often combined with other rehabilitation methods, such as motor imagery, conventional physical therapy, and neuromuscular electrical stimulation. Additionally, existing studies mostly focus on peripheral nerve injuries of the upper limbs and facial nerve injuries, while there is a significant lack of exploration of peripheral nerve injuries of the lower limbs. This may be related to the fact that the lower limb nerves are more surrounded by abundant muscle tissues and are relatively less prone to damage.

Based on the current evidence, we believe that MT should be combined with conventional rehabilitation and specific techniques rather than used alone. For limb nerve injuries, it is recommended to combine MT with neuromuscular electrical stimulation (NMES) to simultaneously regulate the central and peripheral pathways; for facial nerve injuries, the combination of MT and MI may be more effective.

The intervention timing of mirror therapy should follow the biological laws of nerve regeneration and functional recovery and be dynamically adjusted. In the early postoperative period (acute/subacute phase), the main goal of intervention should be to prevent acquired disuse, maintain the activity of the cortical functional area on the affected side, and provide positive psychological feedback. The action design should mainly be painless and simple movements of the healthy side leading the affected side to imitate. After the recovery period, the treatment focus should shift to reconstructing functional motor control and sensory integration. At this time, the main goal of MT is the patient’s daily living activities ability and the ability related to occupational demands.

## Discussion

### Changes in the body after peripheral nerve injury

Following peripheral nerve injury, Wallerian degeneration occurs in the axons. Schwann cells and macrophages collaborate to break down and clear the damaged axons and myelin debris, thereby activating an inflammatory cascade. The proliferating and differentiating Schwann cells secrete various neurotrophic factors and form Büngner bands, which provide directional guidance and a regenerative microenvironment for axonal regeneration, thereby promoting nerve repair and regeneration (38–40).

At the spinal level, the interruption of sensory input leads to a state of hyperexcitability in the dorsal horn neurons, accompanied by a reduction in inhibitory function. Microglia and astrocytes are activated and release pro-inflammatory cytokines. Together, these mechanisms sustain and amplify pain signals (41).

Concurrently, these alterations immediately trigger extensive remodeling within the central nervous system, resulting in a reorganization of the cortical functional map (42). This is manifested as reduced activity in the sensorimotor cortical areas corresponding to the damaged nerve due to impaired afferent signaling, and the cortical representation of these areas is encroached upon by adjacent functional regions or the contralateral hemisphere (43, 44). While this central remodeling may initially hold some compensatory significance, if it persists abnormally, it can impede effective output from the damaged side and hinder functional recovery. During cortical remodeling, the release of various neurotrophic factors, transported via descending pathways, indirectly supports Schwann cell activity and axonal regeneration.

Peripheral nerve injury constitutes a “bottom-up” insult. Therefore, nerve repair can be approached through rehabilitation that combines both peripheral and central interventions. By activating remodeling within the central nervous system and indirectly modulating Schwann cells and neurotrophic factors, corresponding axonal regeneration and functional recovery of the peripheral nerve can be improved.

### Advantages of MT

MT is straightforward to administer, safe, non-invasive, and free of adverse effects. It can be introduced in the early stages of the condition and has shown favorable cost-effectiveness. As an effective auxiliary intervention, it is particularly suitable for regions with limited healthcare resources and for long-term home-based rehabilitation. It is recommended to adopt the model of “structured clinical treatment + standardized family training.” For the clinical treatment part, it is suggested to conduct 3–5 sessions per week, each session including 15–30 min of specialized MT integration training. For the family training part, a clear and safe daily family training plan should be formulated for the patients, with each session lasting 15–20 min. The popularization of family training can extend the treatment scenarios, consolidate the therapeutic effects, and enhance the patients’ active participation.

### Research limitations and future directions

Limitations of this review include the lack of a detailed elucidation of the specific molecular mechanisms and physiological processes underlying the central regulatory effects of mirror therapy. Furthermore, the number of high-quality randomized controlled trials (RCTs) included is limited, and there is considerable heterogeneity among studies in terms of intervention subjects, intervention methods, duration, and outcome measures.

Future research should aim to conduct more rigorously designed, large-sample RCTs to identify the optimal timing for mirror therapy intervention, determine the duration of its therapeutic effects, and optimize combined intervention strategies to enhance overall rehabilitation outcomes. The integration of neuroimaging and neurophysiological techniques such as fMRI, functional near-infrared spectroscopy (fNIRS) and electroencephalogram (EEG) may also help identify biomarkers predictive of treatment response, thereby providing more direct evidence regarding the central mechanisms of mirror therapy.

## Conclusion

MT, as a form of central nervous system modulation, has demonstrated certain therapeutic effects on both motor and sensory functions in patients with peripheral nerve injuries. It is characterized by simplicity, cost-effectiveness, and non-invasiveness, making it particularly suitable for low-cost and long-term home-based rehabilitation. Future research should focus on elucidating the molecular mechanisms underlying the central regulatory effects of mirror therapy, conducting well-designed clinical trials with large sample sizes, and exploring its efficacy under various combined intervention strategies, thereby providing more robust evidence for the application of MT.
